# Health-Related Quality of Life and Stress-Related Disorders in Patients with Complicated Diverticular Disease under Conservative Management

**DOI:** 10.3390/healthcare11101383

**Published:** 2023-05-10

**Authors:** Tudor Mateescu, Bogdan Miutescu, Alin Nicola, Cristian Oancea, Paula Irina Barata, Cristi Tarta, Lazar Fulger, Cristian Paleru

**Affiliations:** 1 Department of General Surgery, “Victor Babes” University of Medicine and Pharmacy Timisoara, Eftimie Murgu Square 2, 300041 Timisoara, Romania; tudor.mateescu@umft.ro (T.M.); tarta.cristi@umft.ro (C.T.); lazarfulger@yahoo.com (L.F.); 2 Doctoral School, “Victor Babes” University of Medicine and Pharmacy Timisoara, Eftimie Murgu Square 2, 300041 Timisoara, Romania; alinnicola.nicola@gmail.com; 3 Department of Gastroenterology and Hepatology, “Victor Babes” University of Medicine and Pharmacy Timisoara, Eftimie Murgu Square 2, 300041 Timisoara, Romania; 4 Department of Thoracic Surgery, “Victor Babes” University of Medicine and Pharmacy Timisoara, Eftimie Murgu Square 2, 300041 Timisoara, Romania; 5 Center for Research and Innovation in Precision Medicine of Respiratory Diseases, “Victor Babes” University of Medicine and Pharmacy Timisoara, Eftimie Murgu Square 2, 300041 Timisoara, Romania; oancea@umft.ro (C.O.); barata.paula@student.uvvg.ro (P.I.B.); 6 Department of Thoracic Surgery, “Carol Davila” University of Medicine and Pharmacy, Bulevardul Eroii Sanitari 8, 050474 Bucuresti, Romania; cpaleru@gmail.com

**Keywords:** quality of life, health-related quality of life, diverticular diseases, psychological stress

## Abstract

Diverticular disease is a common gastrointestinal disorder with increasing prevalence in advanced age. This study aimed to investigate the impact of age and complexity of diverticulitis on health-related quality of life (HRQoL) and stress-related disorders. A cross-sectional study was conducted on 180 patients, including adults (18–64 years) with complicated diverticular disease, the elderly (≥65 years) with complicated diverticular disease, and a control group with uncomplicated symptomatic diverticular disease. HRQoL and stress-related disorders were assessed using the SF-36, GIQLI, HADS, and PHQ-9 questionnaires at baseline and six months after the initial episode of diverticulitis. At diagnosis, the adult group had significantly lower mean physical and mental scores compared with the elderly and control groups (*p* < 0.001). At the 6-month follow-up, the mean physical score increased for all groups, but the difference between adults and the elderly remained significant (*p* = 0.028). The adult group had a significantly lower mean GIQLI score at diagnosis compared with the elderly and control groups (*p* < 0.001), although after 6 months it increased and the difference became insignificant. Anxiety scores at diagnosis were significantly higher in the adult group compared with the control group (*p* = 0.009). The complexity of diverticulitis and age significantly impacted HRQoL at diagnosis, with adults having lower physical and mental scores compared with elderly patients and controls. Although improvements were observed after 6 months, the difference between adults and the elderly remained significant for physical HRQoL scores. This highlights the need for tailored management strategies and psychosocial support to optimize patient outcomes across age groups and diverticulitis complexity.

## 1. Introduction

Diverticular disease encompasses a range of clinical presentations, from asymptomatic diverticulosis to symptomatic diverticulitis, characterized by the presence of diverticula in the colon. Diverticula are small, pouch-like protrusions of the colonic mucosa and submucosa through the muscularis propria layer [1]. The prevalence of the diverticular disease has been increasing over the past few decades, making it a significant public health concern. Understanding the epidemiology and risk factors of this condition is crucial for the development of effective prevention and management strategies [2].

Diverticular disease can be classified into three categories: asymptomatic diverticulosis, symptomatic uncomplicated diverticular disease (SUDD), and complicated diverticular disease, also described as acute diverticulitis [3]. Asymptomatic diverticulosis refers to the presence of diverticula without any clinical symptoms. SUDD is characterized by intermittent abdominal pain and discomfort without signs of inflammation [4]. Acute diverticulitis, the most severe form, involves the inflammation or infection of the diverticula, which may lead to complications such as abscesses, perforation, or fistula formation. Diverticular disease is a global health issue with considerable variation in prevalence across different populations. In Western countries, the prevalence of diverticulosis is estimated to be around 30–50% in individuals aged 60 years and older, whereas, in Asian countries, the prevalence is comparatively lower, at approximately 10–20% in the same age group [5]. The incidence of diverticulitis is also on the rise, with a reported increase of 50% in hospitalizations due to diverticulitis [6].

Several risk factors have been associated with the development and progression of diverticular disease. These factors can be broadly classified into modifiable and non-modifiable risk factors [7]. Low-fiber diets have been consistently linked to an increased risk of diverticular disease. A prospective cohort study found that individuals consuming less than 14 g of fiber per day had a 50% higher risk of developing diverticulitis compared with those consuming more than 25 g per day [8]. Obesity is a significant risk factor for diverticular disease, particularly for the development of complicated diverticulitis. A meta-analysis revealed that obese individuals had a more than 70% higher risk of diverticulitis compared with those with a normal body mass index (BMI) [9]. Sedentary lifestyles are associated with an increased risk of diverticular disease. Regular physical activity, particularly vigorous activity, has been shown to reduce the risk of diverticulitis by 25% [10]. The risk of developing the diverticular disease increases with age, with the majority of cases occurring in individuals aged 60 years and older [11]. Additionally, familial aggregation of diverticular disease suggests a genetic component to its development.

Diverticular disease encompasses a spectrum of conditions that can significantly impact a patient’s quality of life (QoL) and psychological well-being [12,13,14]. Symptomatic uncomplicated diverticular disease (SUDD) is characterized by abdominal pain and discomfort without overt inflammation. Despite its milder presentation compared with complicated diverticular disease, SUDD can significantly impair patients’ QoL. On the other hand, acute diverticulitis and its complications, such as abscesses, perforation, or fistula formation, can severely affect patients’ QoL [15]. Studies described that patients with complicated diverticulitis had significantly lower QoL scores in the physical and mental components of the SF-36 questionnaire compared with patients with uncomplicated diverticulitis. Additionally, patients with complicated diverticulitis had higher rates of re-hospitalization and longer recovery times, further contributing to diminished QoL [16,17,18].

The relationship between diverticular disease and psychological disorders, such as anxiety and depression, has been increasingly recognized. It is hypothesized that elderly patients with complicated diverticular disease experience a greater negative impact on health-related quality of life (HRQoL) and a higher prevalence of stress-related disorders compared with younger patients with complicated diverticular disease. The current study aims to assess and compare the HRQoL in elderly and younger adult patients with complicated diverticular disease using validated questionnaires, such as the Short Form-36 (SF-36) and the Gastrointestinal Quality of Life Index (GIQLI). A secondary aim is to evaluate the prevalence of stress-related disorders, including anxiety and depression, in patients with complicated diverticular disease using validated screening tools such as the Hospital Anxiety and Depression Scale (HADS) or the Perceived Stress Scale (PSS-10). Additionally, we aim to explore potential demographic, clinical, and psychosocial factors associated with HRQoL and stress-related disorders in patients with complicated and uncomplicated diverticular disease, including age, sex, comorbidities, and social support.

## 2. Materials and Methods

### 2.1. Study Design and Ethical Considerations

A cross-sectional study was designed at the Clinical Emergency Hospital “Pius Brinzeu” in Timisoara, Romania. The study was conducted according to the guidelines of the Declaration of Helsinki. The researchers involved in the current study gathered background and medical data from the hospital database and the associated patients’ paper records, where all treatments, procedures, and demographics were registered.

The inclusion criteria comprised the following particularities: (1) at least 18 years old; (2) a diagnosis of complicated diverticular disease proven by computed tomography (CT); (3) no previous episodes of complicated diverticular disease; and (4) a diagnosis of uncomplicated diverticular disease, for the control group. Patients were excluded for incomplete medical records, lack of consent identified from the personal paper records, previous diagnosis of psychiatric disorders, or class 3 or 4 acute diverticulitis based on the Hinchey classification [19].

The sample size was determined using a convenience sampling method. It was calculated that 192 cases represent the ideal sample size, considering an approximated prevalence of symptomatic diverticular disease at an average of 10% for advanced age [20], a margin of error of 5%, and a confidence level of 95%. The threshold for statistical significance was 0.05. The statistical power (1-β) calculation was 80% for a type I error rate of 5%. A total of 300 patients were surveyed, of which 224 were accepted to participate. The surveys were completed online with the help of physicians involved in the study. A total of 218 questionnaires were successfully completed, and 180 were included in the final analysis after excluding those with incomplete medical records.

In this study, we enrolled three groups of patients to investigate the impact of age and complexity of diverticulitis on psychosocial outcomes. The first group consisted of adults (aged 18–64 years) with complicated diverticulitis stage 1b (abscess <5 cm in the proximity of primary inflammation), and stage 2 (intra-abdominal, pelvic or retroperitoneal abscess, or abscess distant from the primary inflammation), according to the Hinchey classification. The second group included elderly patients (aged 65 years or older) with complicated diverticulitis (stages 1b, and 2). We chose this age cutoff based on the definition of elderly by the World Health Organization. The third group was a control group of patients with uncomplicated diverticulitis, which was defined as diverticulitis without the presence of an abscess, fistula, perforation, or obstruction, and includes stage 0 (clinically mild diverticulitis) and stage Ia (pericolic inflammation). All patients in the three groups were recruited from the same tertiary care hospital, and their diagnoses were confirmed by radiologic imaging.

### 2.2. Questionnaires and Variables

The current study assessed the quality of life of the patients at baseline during hospital admission and six months after the initial episode of diverticulitis, as well as the presence of persistent complaints, such as abdominal pain, bloating, and altered bowel habits. To assess the HRQoL and stress-related disorders in patients with complications, we used the SF-36, GIQLI, HADS, and PHQ-9 questionnaires.

The Short Form-36 (SF-36) is a widely used scale for measuring health-related quality of life (HRQOL) and functional status in both clinical and research settings. It consists of 36 items that assess eight domains of HRQOL: physical functioning, role limitations due to physical health problems, bodily pain, general health perceptions, vitality, social functioning, role limitations due to emotional problems, and mental health. The SF-36 is a self-reported questionnaire that asks respondents to rate their health status over the previous four weeks. Each domain is scored from 0 to 100, with higher scores indicating better health status and quality of life. The scores can be aggregated to produce two summary scores: the Physical Component Summary (PCS) and the Mental Component Summary (MCS). The SF-36 has been extensively validated and is available in multiple languages [21].

The Gastrointestinal Quality of Life Index (GIQLI) scale is a health-related quality of life questionnaire that assesses the impact of gastrointestinal diseases on an individual’s quality of life [22]. It is a disease-specific measure and is designed to capture the impact of gastrointestinal symptoms on daily activities and well-being. The GIQLI consists of 36 items that cover 5 domains of gastrointestinal symptoms and their impact on daily life: gastrointestinal symptoms, physical function, emotional function, social function, and medical treatment. Respondents rate the frequency and severity of symptoms, the extent to which symptoms interfere with daily activities, and their overall satisfaction with their gastrointestinal health. The GIQLI is scored on a 7-point Likert scale, with higher scores indicating the better health-related quality of life. The total score ranges from 0 to 144, with higher scores indicating better quality of life. The GIQLI has been extensively validated and has been shown to be reliable and valid in measuring the impact of gastrointestinal diseases on quality of life.

The Hospital Anxiety and Depression Scale (HADS) is a self-report scale designed to measure anxiety and depression in individuals who are being treated in a hospital or outpatient setting. It is a widely used screening tool for identifying individuals with anxiety and depression symptoms. The HADS consists of 14 items, 7 of which assess anxiety symptoms (HADS-A) and 7 of which assess depression symptoms (HADS-D) [23]. Each item is scored on a 4-point scale, with higher scores indicating greater levels of anxiety or depression. The HADS does not include questions about physical symptoms, which helps to distinguish anxiety and depression from physical illness.

The Perceived Stress Scale (PSS-10) is a widely used self-report questionnaire designed to measure the degree to which individuals perceive situations in their lives as stressful [24]. The PSS-10 consists of 10 items, with each item rated on a 5-point Likert scale ranging from 0 (never) to 4 (very often). Participants are asked to indicate how often they have felt or thought a certain way in the past month. The items capture various aspects of perceived stress, including feelings of helplessness, lack of control, and inability to cope with daily life demands. The scores of the individual items are summed up to generate a total score, which ranges from 0 to 40. Higher scores indicate higher levels of perceived stress. The PSS-10 has been found to be a reliable and valid instrument with good psychometric properties in various populations and settings.

### 2.3. Statistical Analysis

GraphPad Prism for Microsoft Windows, version 6.07, was used to conduct the statistical analysis (GraphPad Software Inc., San Diego, CA, USA). The Kolmogorov–Smirnov test was used to assess the normality of the data. The mean value, which represents central tendency, and the standard deviation, which measures dispersion, were used to represent normally distributed data. The ANOVA test was used to examine the difference in means between the three comparison groups, while the Student’s *t*-test was performed to compare two groups presented using the mean and standard deviation. The median and interquartile range (IQR) were used to characterize non-normally distributed data, presented in box plots, while the Kruskal–Wallis test was used to compare these variables. Considering the frequency assumption for the Chi-square test was not fulfilled, proportions were compared using Fisher’s exact test. A *p*-value below 0.05 was regarded as statistically significant.

## 3. Results

### 3.1. Background Analysis

A total of 75 adult patients with complicated diverticular disease were included in the final analysis, as well as 53 elderly patients with acute diverticulitis and 52 controls with uncomplicated symptomatic diverticular disease. The background analysis presented in Table 1 highlights several variables, including age, gender, area of residence, smoking status, pack-year smoking, the number of patients with obesity, patients who admitted having a low-fiber diet and chronic constipation, as well as the Charlson Comorbidity Index (CCI) score. The mean age of the adult group was 52.1 ± 9.0 years, while the elderly group’s mean age was 69.6 ± 3.8 years. The control group had a mean age of 63.7 ± 8.5 years. There was a statistically significant difference in age between the groups (*p* < 0.001). The percentage of females in the adult, elderly, and control groups were 55.7%, 61.7%, and 54.3%, respectively, with no statistically significant difference between the groups (*p* = 0.502).

There was no statistically significant difference between the groups for the urban area of residence (*p* = 0.902), with 51.0% in the adult group, 48.6% in the elderly group, and 51.4% in the control group. No significant difference was observed in smoking status (*p* = 0.775) or pack-year smoking (*p* = 0.671) between the groups. The prevalence of obesity was similar across the groups, with 28.2% in the adult group, 27.1% in the elderly group, and 31.4% in the control group, with no statistically significant difference between them (*p* = 0.766). Additionally, no statistically significant difference was observed in the prevalence of low-fiber diet between the groups (*p* = 0.923). The prevalence of chronic constipation was 19.5% in the adult group, 22.4% in the elderly group, and 25.7% in the control group, with no statistically significant difference between them (*p* = 0.496). Lastly, no significant difference was observed between the groups for Charlson Comorbidity Index scores greater than 3 (*p* = 0.110), with 13.4% in the adult group, 23.4% in the elderly group, and 20.0% in the control group.

### 3.2. Diverticular Disease Characteristics

Table 2 presents the characteristics of diverticular disease among the three study groups. The median bowel movements per day were 2.0 (IQR: 1–5) for adults, 1.0 (IQR: 0–4) for the elderly, and 1.5 (IQR: 0–2) for the control group. The difference between the groups was not statistically significant (*p* = 0.206). The number of days per week of loose bowel movements was 3.0 (IQR: 1–6) for adults, 2.0 (IQR: 0–5) for the elderly, and 1.0 (IQR: 0–3) for the control group. The difference was not statistically significant (*p* = 0.148). The number of days per week of hard bowel movements was 2.0 (IQR: 0–3) for adults, 3.0 (IQR: 1–5) for the elderly, and 4.0 (IQR: 1–7) for the control group. The difference was not statistically significant (*p* = 0.227). The number of days per week of pain was significantly higher in adults (4.0, IQR: 1–7) and the elderly (4.5, IQR: 2–7) than in the control group (2.0, IQR: 0–3) (*p* = 0.030).

The Hinchey classification was not significantly different between adults and the elderly (*p* = 0.158). The majority of patients in all groups were under Hinchey 1b classification (74.7% in adults vs. 62.3% in the elderly). The treatment options were significantly different between the groups, since intravenous (IV) antibiotics were given to 81.2% of adults, 87.9% of the elderly, and 17.1% of the control group. Oral antibiotics were given to 24.2% of adults, 20.6% of the elderly, and 70.5% of the control group. Anti-inflammatory drugs were given to 53.0% of adults, 41.1% of the elderly, and 64.8% of the control group. A liquid diet was given to 58.4% of adults, 64.5% of the elderly, and 21.9% of the control group. Stool softeners were given to 51.0% of adults, 67.3% of the elderly, and 76.2% of the control group. IV fluids were given to 65.8% of adults, 75.7% of the elderly, and 37.1% of the control group. The *p*-values for the comparison between adults and the elderly were calculated for each treatment option. IV antibiotics, oral antibiotics, and anti-inflammatory drugs showed significant differences (*p* < 0.05). However, liquid diet, stool softeners, and IV fluids did not show significant differences (*p* > 0.05).

### 3.3. Analysis of Standardized Questionnaires

**Figure 1:**
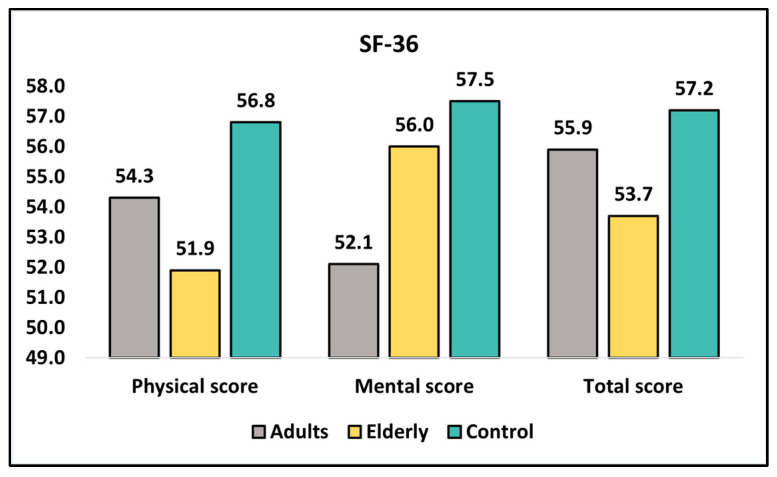
Analysis of SF-36 questionnaire at diagnosis.

At diagnosis, the mean physical score for adults was 54.3 ± 7.6, while the elderly group had a slightly lower mean score of 51.9 ± 8.0, and the control group had the highest mean score of 56.8 ± 7.3. The difference between the groups was statistically significant, with a *p*-value of <0.001. When comparing only adults and the elderly, the *p*-value was 0.015, indicating a significant difference between these two groups. The mental score at diagnosis showed a similar pattern, with the adult group having a mean score of 52.1 ± 8.1, the elderly group having a mean score of 56.0 ± 6.7, and the control group having a mean score of 57.5 ± 6.9, as seen in Figure 1. The difference between the groups was also statistically significant (*p*-value < 0.001), and the difference between adults and the elderly remained significant with a *p*-value of <0.001. For the total SF-36 score at diagnosis, the adult group had a mean score of 55.9 ± 8.4, the elderly group had a mean score of 53.7 ± 7.4, and the control group had a mean score of 57.2 ± 6.4. The difference between the groups was statistically significant (*p*-value < 0.001), and the difference between adults and the elderly was also significant, with a *p*-value of 0.030.

At the 6-month follow-up, the mean physical score for adults increased to 55.9 ± 7.2, the elderly group had a mean score of 53.8 ± 8.0, and the control group had a mean score of 57.2 ± 6.8. The difference between the groups remained statistically significant (*p*-value < 0.001), and the difference between adults and the elderly was also significant, with a *p*-value of 0.028. The mental score at the 6-month follow-up also increased for all groups: adults had a mean score of 55.2 ± 8.5, the elderly group had a mean score of 56.6 ± 6.9, and the control group had a mean score of 58.4 ± 7.3. The difference between the groups was statistically significant (*p*-value = 0.005), but the difference between adults and the elderly was not significant (*p*-value = 0.161). Lastly, the total SF-36 score at the 6-month follow-up showed an increase for all groups: adults had a mean score of 56.7 ± 8.0, the elderly group had a mean score of 55.9 ± 7.8, and the control group had a mean score of 57.7 ± 7.5. The difference between the groups was not statistically significant (*p*-value = 0.243), and the difference between adults and the elderly was also not significant (*p*-value = 0.426), as seen in Table 3.

In our paired comparison analysis, we observed significant improvements in both physical and mental scores for the adults group (*n* = 75) at the 6-month follow-up compared with their scores at diagnosis. The physical scores increased by a mean difference of 1.6 (*p*-value = 0.013), while the mental scores improved by a mean difference of 3.1 (*p*-value < 0.001). For the elderly group (*n* = 53), we found a significant improvement in their physical scores, with a mean difference of 1.9 (*p*-value = 0.027), and total scores, with a mean difference of 2.2 (*p*-value = 0.012). However, there was no significant change in their mental scores. Notably, there were no significant changes in any of the scores for the control group (*n* = 52). These findings suggest that patients with diverticular disease, particularly in the adults and elderly groups, experienced improvements in physical and mental health aspects over the course of 6 months, indicating a positive response to the treatment or management strategies employed.

At diagnosis, the mean GIQLI score for the adult group was 118 ± 14.6, while the elderly group had a lower mean score of 111 ± 12.2, and the control group had the highest mean score of 124 ± 10.8. The difference between the groups was statistically significant, with a *p*-value of <0.001. When comparing only adults and the elderly, the *p*-value was <0.001, indicating a significant difference between these two groups. The median GIQLI score at diagnosis showed a similar pattern. The adult group had a median score of 120 with an interquartile range (IQR) of 104–136, the elderly group had a median score of 113 with an IQR of 98–125, and the control group had a median score of 128 with an IQR of 109–131. The difference between the groups was statistically significant (*p*-value <0.001), and the difference between adults and the elderly remained significant with a *p*-value of <0.001.

At the 6-month follow-up, the mean GIQLI score for adults increased to 125 ± 12.7, the elderly group had a mean score of 122 ± 13.5, and the control group had a mean score of 129 ± 12.0. The difference between the groups remained statistically significant (*p*-value <0.001), but the difference between adults and the elderly was not significant (*p*-value 0.070). The median GIQLI score at the 6-month follow-up also increased for all groups: adults had a median score of 124 with an IQR of 109–132, the elderly group had a median score of 115 with an IQR of 106–128, and the control group had a median score of 131 with an IQR of 114–138. The difference between the groups was statistically significant (*p*-value <0.001), and the difference between adults and the elderly remained significant with a *p*-value of <0.001, as described in Table 4.

**Figure 2:**
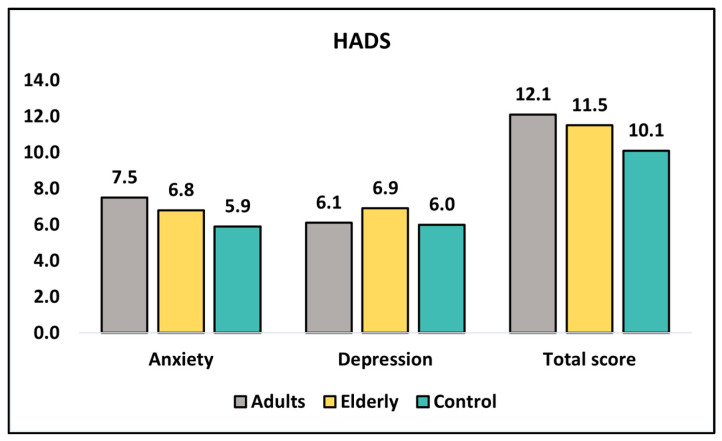
Analysis of HADS questionnaire at diagnosis.

Table 5 presents the results of an analysis of the HADS questionnaire for three groups of participants at diagnosis and six months after the initial survey. The HADS questionnaire is a measure of anxiety and depression, where higher scores indicate greater levels of anxiety or depression. The table presents the mean scores for anxiety, depression, and total scores at diagnosis and at six months, as well as the *p*-value for each comparison. At diagnosis, the anxiety score for the group of adults was significantly higher than that of the control group, with a *p*-value of 0.009. However, there were no significant differences in anxiety scores between any of the groups for the elderly group or the total score, as seen in Figure 2. Additionally, there were no significant differences in depression scores between any of the groups at diagnosis. At 6 months, there were no significant differences in anxiety scores between any of the groups. There were also no significant differences in depression scores between any of the groups, except for a marginally significant difference between the adults and elderly groups, with a *p*-value of 0.157.

**Figure 3:**
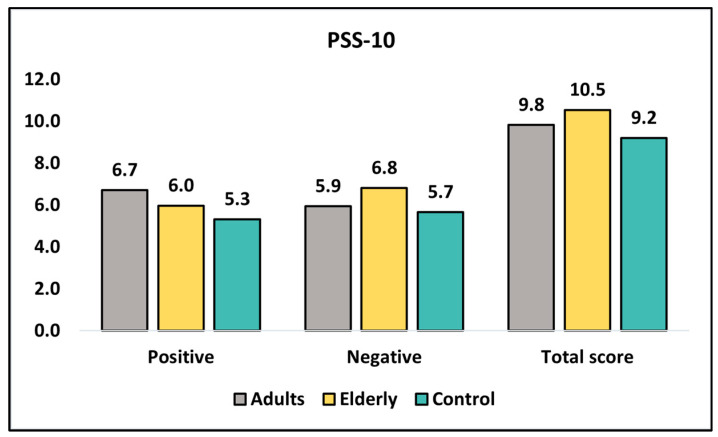
Analysis of PSS-10 questionnaire at diagnosis.

At diagnosis, Table 6 shows that adults had a higher mean score for positive perceived stress (6.71) compared with the elderly (5.97) and control (5.32) groups (Figure 3). This difference was statistically significant (*p* = 0.020). On the other hand, for negatively perceived stress, the elderly group had a higher mean score (6.81) compared with the adults (5.94) and control (5.66) groups, and this difference was also statistically significant (*p* = 0.007). However, the total score did not differ significantly among the three groups (*p* = 0.125). At the 6-month follow-up, there was no significant difference in positive perceived stress scores among the three groups (*p* = 0.201), although the elderly group had the highest mean score (7.09). For negatively perceived stress, the elderly group had a higher mean score (6.14) compared with the adults (5.44) and control (5.28) groups, but the difference was not statistically significant (*p* = 0.124). The total score did not differ significantly among the three groups (*p* = 0.134). Overall, there were significant differences in perceived stress scores between adults and elderly groups at diagnosis for negatively perceived stress (*p* = 0.022) and at the six-month follow-up for positive perceived stress (*p* = 0.006).

## 4. Discussion

### 4.1. Literature Findings

The current study found that adults and elderly patients with a complicated diverticular disease under conservative management experienced significantly more days per week of pain compared with the control group. Treatment options were significantly different between the groups, with IV antibiotics, oral antibiotics, and anti-inflammatory drugs showing significant differences between adults and the elderly. The results of the study suggest that both adults and elderly patients had lower HRQoL compared with the control of patients with uncomplicated symptomatic diverticular disease at diagnosis. Although younger adults with acute diverticulitis had a significantly higher physical component of HRQoL compared with the elderly group, the latter had better mental scores. While physical health had improved for all groups at six months, there were still differences between the groups in terms of physical health compared with the control group, where elderly patients had the lowest scores. However, there were no significant differences in mental or total scores between any of the groups at six months. In summary, the GIQLI questionnaire results indicate significant differences in the mean and median scores at diagnosis between adults, the elderly, and control groups. At the 6-month follow-up, improvements were observed in all groups, but the differences between the groups remained significant. The difference between adults and elderly in mean scores was not significant at the 6-month follow-up, while the difference in median scores remained significant.

Regarding the SF-36 scale used in patients with diverticular disease, in one Italian study, the researchers examined the quality of life (QoL) in patients with uncomplicated symptomatic diverticular disease using the SF-36 questionnaire [25]. They found that the QoL of patients with the diverticular disease was significantly impaired compared with the Italian normative group, with no notable differences between genders or age groups (with a cut-off of 65 years). The study aimed to investigate the effects of two treatment regimens, rifaximin (a non-absorbable antibiotic) and mesalazine (an anti-inflammatory), on QoL [26]. Patients were randomly assigned to one of these treatments, and after six months of therapy, improvements were observed in the mean scores of almost all domains of the SF-36 questionnaire for both physical and mental performance. No significant differences were found between the two treatment groups in mean QoL scores and mean Global Symptom Score (GSS) at baseline. The study concurred with previous findings that cyclic therapy with either an anti-inflammatory or a non-absorbable antibiotic effectively relieved symptoms in patients with DD. After six months of treatment, mesalazine proved to be as effective as rifaximin in reducing the mean GSS, with mesalazine-treated patients showing a lower GSS than those treated with rifaximin. Both treatment groups experienced improvements in mean scores across almost all SF-36 domains, indicating significant improvements in physical and mental status. Mesalazine-treated patients exhibited higher mean SF-36 scores in nearly all domains compared with rifaximin-treated patients after six months of therapy, with a marked improvement in physical status.

A recent study established a survey designed for patients with diverticular disease [27]. In the study, researchers were the first to establish the Minimal Clinically Important Difference (MCID) and Patient-Acceptable Symptom State (PASS) for the Diverticular Disease Quality of Life (DV-QOL) measure. The researchers analyzed prospectively collected data from a diverse group of patients with diverticular disease and identified a score of 3.2 out of 10 as the PASS threshold, distinguishing patients with HRQoL-impacting diverticulitis from those without. A change of 2.2 points in the DV-QOL was determined to be the most suitable MCID, as it exceeded distribution-based MCIDs and aligned with patients’ perceptions of significant change. The study also found that patients with HRQoL-limiting disease at baseline were more likely to be younger and male, consistent with previous findings identifying younger age and male sex as risk factors for recurrence. However, other studies reported no differences in HRQoL between patients’ sex and age groups. When utilizing DV-QOL as an outcome measure in future studies, researchers should take into account how these factors might influence HRQoL trajectories or treatment impacts. The study also observed that patients with worse DV-QOL frequently had Medicaid as their primary insurance and lower educational levels. The results were meant to be descriptive and to support future hypothesis-generating research to investigate the cause of HRQoL-limiting disease and the association between patient characteristics and DV-QOL trends.

Another study aimed to develop a validated clinical score for symptomatic uncomplicated diverticular disease (SUDD) following an acute diverticulitis (AD) episode [28]. Researchers used data from a previous prospective study of patients after AD to create the initial version of the score, which was then validated using a focus group of patients after AD SUDD through structured cognitive, personal interviews. The Diverticular Clinical Score (DICS) was applied to a second validation cohort, and the scores were compared with physicians’ global assessments of disease severity and inflammatory markers. A strong correlation was found between the total questionnaire score and the presence of elevated inflammatory markers. The mean score for patients with elevated inflammatory markers was significantly higher than for those without inflammation (17.8 vs. 6.2). The DICS demonstrated high internal consistency (Cronbach’s α = 0.91) and accurately discriminated between patients with and without active disease, as measured by the physician’s global assessment (AUROC = 0.989). The researchers concluded that the newly developed DICS accurately and reproducibly quantified SUDD-related symptom severity, and it might be useful for monitoring SUDD in clinical practice and research, as well as facilitating patient stratification and therapeutic decisions.

A German study evaluated a stratification of different types of diverticular disease in terms of course and treatment [29]. The primary endpoint was the rate of recurrence within a 2-year follow-up, with the secondary outcome measures GIQLI, SF-36 quality of life, frequency of gastrointestinal complaints, and postoperative complications. The results showed that after conservative management, 40% of type 1b patients required surgery for recurrence compared with 80% of type 2a/b patients. Among type 2a patients with micro-abscess, 60% needed surgery for recurrence, while 100% of type 2b patients with macro-abscess did. Type 2a patients had higher GIQLI scores than type 2b patients and higher scores on the SF-36 MCS scale. Lastly, type 3b patients with recurrent diverticulitis without complications experienced less painful constipation when they underwent surgery compared with conservative treatment. In conclusion, differentiating between type 2a and 2b based on abscess size appeared to have a significant difference in terms of quality of life, as type 2b patients required surgery while type 2a patients could be treated conservatively.

In a study examining anxiety, depression, and non-gastrointestinal symptoms in irritable bowel syndrome (IBS) and diverticular disease, researchers modified the Patient Health Questionnaire 15 (PHQ-15) by excluding three gastrointestinal items to create the PHQ-12 Somatic Symptom (PHQ-12 SS) scale [30]. The objective was to compare the value of the PHQ-12 SS scale with the Hospital Anxiety and Depression (HAD) scale in predicting symptoms and patient behavior in IBS and diverticular disease. The results showed that the PHQ-12 SS scores for IBS and symptomatic diverticular disease patients were significantly higher than those of healthy volunteers. ROC curves revealed that a PHQ-12 SS score greater than 6 provided a sensitivity for IBS of 66.4%, a specificity of 94.7%, and a positive likelihood ratio (PLR) of 13.2. This performance was significantly better than that associated with a HADS anxiety score greater than 7 (PLR = 3.0) and a depression score greater than 7 (PLR = 6.5). The PHQ-12 SS scale showed a strong correlation between IBS severity and general practitioner visits in both IBS and diverticular disease patients. Therefore, the PHQ-12 SS scale proved to be a useful clinical tool for assessing patient behavior with symptomatic diverticular disease, similar to our study findings.

Regarding the follow-up and long-term evaluation of patients with diverticular disease, which was one of the current study objectives, another study aimed to review evidence on long-term outcomes after diverticular surgery for diverticulosis/diverticulitis, including health-related quality of life (HRQoL), functional disorders, abdominal pain, and patient satisfaction [31]. The results indicated that HRQoL improved in most cases after surgery, and patient satisfaction was generally high. However, chronic abdominal pain and functional disorders were present in a significant portion of patients. Despite this, functional disorders did not result in decreased HRQoL in most studies, and no increase in functional disorders was observed after elective diverticular surgery in longitudinal analyses. The study concluded that it is essential to carefully discuss functional disorders with patients before surgery and to consider a thorough clinical assessment, including incontinence scoring.

### 4.2. Study Limitations and Future Perspectives

Several limitations of the present study should be acknowledged. This study utilized a convenience sampling method, which might limit the generalizability of the findings to the broader population; thus, a stratified random sampling or cluster sampling methods could be more appropriate for future studies. The cross-sectional nature of the study design cannot establish the causal relationships between the variables of interest, which could be a potential aim for future studies. Thus, longitudinal or experimental designs could be more appropriate to determine the causality of the observed associations. The assessment of the patient’s quality of life, persistent complaints, and other symptoms relied on self-reported measures, which might be subject to recall bias. This limitation could affect the accuracy of the reported data and the validity of the study findings. Additionally, the study was conducted in a single tertiary care hospital, which might limit the generalizability of the findings to other healthcare settings or geographic regions. A multi-center study could provide a more comprehensive understanding of the impact of age and the complexity of diverticulitis on outcomes. It is important to mention that some patients were excluded from the study due to incomplete medical records, which can determine a certain degree of selection bias. Additionally, the initial sample size was 300 patients, while only 180 were included in the final analysis after excluding those with incomplete medical records. This loss to follow-up could potentially introduce attrition bias. Nevertheless, the sample size requirements were met. Lastly, even though the majority of *p*-values were not significant at a 0.01 threshold for significance, the statistical power calculation allows for a proper reliability of our findings, considering the sample size requirements were met.

## 5. Conclusions

In conclusion, our study found that patients with complicated diverticular disease, regardless of age, have lower HRQoL scores during the acute event compared with patients with uncomplicated diverticular disease, suggesting that the complexity of diverticulitis has a more significant impact on HRQoL than age. Therefore, clinicians should be mindful of the potential negative impact of complicated diverticular disease on patients’ quality of life and provide appropriate support and care to improve their well-being. Further research is needed to evaluate the long-term effects of complicated diverticular disease on HRQoL and to explore potential strategies to optimize the management and treatment of these patients.

## Figures and Tables

**Table 1:** Background analysis.

Variables	Adults (*n* = 75)	Elderly (*n* = 53)	Control (*n* = 52)	*p*-Value
Age (mean ± SD)	52.1 ± 9.0	69.6 ± 3.8	63.7 ± 8.5	<0.001
Age range	43–64	65–84	51–73	-
Gender (female, %)	42 (55.7%)	33 (61.7%)	28 (54.3%)	0.502
Area of residence (urban, %)	38 (51.0%)	26 (48.6%)	27 (51.4%)	0.902
Smoking status (yes, %)	26 (32.9%)	17 (31.8%)	19 (36.2%)	0.775
Pack-year smoking (median, IQR)	29.5 (22.0–36.5)	31.0 (21.5–37.0)	32.5 (22.0–38.0)	0.671
Obesity (yes, %)	21 (28.2%)	16 (27.1%)	16 (31.4%)	0.766
Low-fiber diet (yes, %)	25 (34.2%)	19 (36.4%)	18 (34.3%)	0.923
Chronic constipation (*n*, %)	15 (19.5%)	12 (22.4%)	14 (25.7%)	0.496
CCI > 3 (*n*, %)	10 (13.4%)	12 (23.4%)	10 (19.2%)	0.110

SD—Standard Deviation; IQR—Interquartile Range; CCI—Charlson Comorbidity Index.

**Table 2:** Characteristics of diverticular disease in the study cohort.

Variables	Adults (*n* = 75)	Elderly (*n* = 53)	Control (*n* = 52)	*p*-Value	*p*-Value *
Bowel movements per day (median, IQR)	2.0 (1–5)	1.0 (0–4)	1.5 (0–2)	0.206	0.729
Days per week of loose bowel movements	3.0 (1–6)	2.0 (0–5)	1.0 (0–3)	0.148	0.544
Days per week of hard bowel movements	2.0 (0–3)	3.0 (1–5)	4.0 (1–7)	0.227	0.281
Days per week of pain	4.0 (1–7)	4.5 (2–7)	2.0 (0–3)	0.030	0.751
Hinchey classification				0.158	0.158
0	-	-	21 (39.3%)		
1a	-	-	20 (37.4%)		
1b	56 (74.7%)	33 (62.3%)	-		
2	19 (26.2%)	20 (37.4%)	-		
Treatment					
IV antibiotics	60 (81.2%)	52 (87.9%)	9 (17.1%)	<0.001	0.152
Oral antibiotics	18 (24.2%)	21 (20.6%)	37 (70.5%)	<0.001	0.497
Anti-inflammatory	39 (53.0%)	22 (41.1%)	34 (64.8%)	0.003	0.060
Liquid diet	43 (58.4%)	34 (64.5%)	12 (21.9%)	<0.001	0.320
Stool softeners	38 (51.0%)	36 (67.3%)	40 (76.2%)	<0.001	0.009
IV fluids	49 (65.8%)	41 (75.7%)	19 (37.1%)	<0.001	0.087

* Calculated between Adults and Elderly; IQR—Interquartile Range; IV—Intravenous.

**Table 3:** Analysis of SF-36 questionnaire at diagnosis and at the six-month follow-up.

SF-36	Adults (*n* = 75)	Elderly (*n* = 53)	Control (*n* = 52)	*p*-Value	*p*-Value *
At diagnosis					
Physical	54.3 ± 7.6	51.9 ± 8.0	56.8 ± 7.3	<0.001	0.015
Mental	52.1 ± 8.1	56.0 ± 6.7	57.5 ± 6.9	<0.001	<0.001
Total score	55.9 ± 8.4	53.7 ± 7.4	57.2 ± 6.4	<0.001	0.030
At 6 months					
Physical	55.9 ± 7.2	53.8 ± 8.0	57.2 ± 6.8	<0.001	0.028
Mental	55.2 ± 8.5	56.6 ± 6.9	58.4 ± 7.3	0.005	0.161
Total score	56.7 ± 8.0	55.9 ± 7.8	57.7 ± 7.5	0.243	0.426

* Calculated between adults and the elderly; SF-36—36-Item Short Form Survey.

**Table 4:** Analysis of GIQLI questionnaire at diagnosis and at the six-month follow-up.

GIQLI	Adults (*n* = 75)	Elderly (*n* = 53)	Control (*n* = 52)	*p*-Value	*p*-Value *
At diagnosis					
Mean (±SD)	118 ± 14.6	111 ± 12.2	124 ± 10.8	<0.001	<0.001
Median (IQR)	120 (104–136)	113 (98–125)	128 (109–131)	<0.001	<0.001
At 6 months					
Mean (±SD)	125 ± 12.7	122 ± 13.5	129 ± 12.0	<0.001	0.070
Median (IQR)	124 (109–132)	115 (106–128)	131 (114–138)	<0.001	<0.001

* Calculated between adults and the elderly; GIQLI—The Gastrointestinal Quality of Life Index; SD—Standard Deviation; IQR—Interquartile Range.

**Table 5:** Analysis of HADS questionnaire at diagnosis and at the six-month follow-up.

HADS	Adults (*n* = 75)	Elderly (*n* = 53)	Control (*n* = 52)	*p*-Value	*p*-Value *
At diagnosis					
Anxiety	7.5 ± 4.3	6.8 ± 4.4	5.9 ± 3.8	0.009	0.204
Depression	6.1 ± 3.5	6.9 ± 3.1	6.0 ± 3.6	0.121	0.059
Total score	12.1 ± 6.0	11.5 ± 5.7	10.1 ± 5.2	0.034	0.421
At 6 months					
Anxiety	6.9 ± 4.7	6.2 ± 4.5	5.8 ± 4.4	0.143	0.232
Depression	6.8 ± 3.1	6.1 ± 4.8	6.0 ± 3.6	0.791	0.157
Total score	10.3 ± 5.5	10.8 ± 6.1	9.7 ± 5.2	0.318	0.493

* Calculated between adults and the elderly; HADS—Hospital Anxiety and Depression Scale.

**Table 6:** Analysis of the PSS-10 questionnaire at diagnosis and at the six-month follow-up.

PSS-10	Adults (*n* = 75)	Elderly (*n* = 53)	Control (*n* = 52)	*p*-Value	*p*-Value *
At diagnosis					
Positive	6.71 ± 3.83	5.97 ± 3.92	5.32 ± 4.08	0.020	0.132
Negative	5.94 ± 2.89	6.81 ± 3.14	5.66 ± 2.17	0.007	0.022
Total score	9.82 ± 4.27	10.54 ± 5.08	9.20 ± 5.13	0.125	0.220
At 6 months					
Positive	6.30 ± 4.12	7.09 ± 4.52	6.11 ± 4.26	0.201	0.006
Negative	5.44 ± 3.10	6.14 ± 3.86	5.28 ± 2.94	0.124	0.109
Total score	9.06 ± 5.28	10.25 ± 6.30	8.86 ± 5.11	0.134	0.102

* Calculated between adults and the elderly; PSS-10—The Perceived Stress Scale.

## Data Availability

The data are available upon request.
